# Study Protocol for the Peruvian Registry of Advanced Heart Failure (REPICAV)

**DOI:** 10.3389/fcvm.2022.896821

**Published:** 2022-05-31

**Authors:** Manuel Chacón-Diaz, Rocío Laymito Quispe, Akram Hernández-Vásquez, Rodrigo Vargas-Fernández

**Affiliations:** ^1^Facultad de Ciencias de la Salud, Universidad Científica del Sur, Lima, Peru; ^2^Instituto Nacional Cardiovascular, EsSalud, Lima, Peru; ^3^Hospital Nacional Alberto Sabogal Sologuren, Lima, Peru; ^4^Centro de Excelencia en Investigaciones Económicas y Sociales en Salud, Vicerrectorado de Investigación, Universidad San Ignacio de Loyola, Lima, Peru

**Keywords:** heart failure, heart disease risk factor, prospective study design, Peru, cardiovascular diseases

## Abstract

**Background:**

Heart failure (HF) is a global problem with a high mortality rate, and advanced HF (AHF) represents the stage with the highest morbidity and mortality. We have no local data on this population and its treatment. The aim of this study will be to determine the epidemiological, clinical, therapeutic, and annual survival characteristics of patients diagnosed with AHF treated in hospitals with HF units in the city of Lima, Peru.

**Methods and Analysis:**

An observational, prospective, multicenter study will be conducted with evaluation at baseline and follow-up at 1, 3, 6, and 12 months after study entry. Patients over 18 years of age with AHF seen in referral health facilities in metropolitan Lima will be included. The cumulative mortality during follow-up will be estimated by the Kaplan-Meier method, and Cox regression models will calculate hazard ratios (HRs) and 95% confidence intervals (CI). Likewise, risk ratio (RR) and 95% CI will be estimated using generalized linear models with binomial family and log link function. This study was approved by the Ethics and Research Committee of the National Cardiovascular Institute (Instituto Nacional Cardiovascular “Carlos Alberto Peschiera Carrillo”—INCOR [in Spanish]; Approval report 46/2021-CEI).

**Discussion:**

In Peru, there are no scientific data on the epidemiology of AHF in the population. This means that physicians are not adequately trained in the characteristics of the Peruvian population to identify patients who could be candidates for advanced therapies and to recognize the optimal time to refer these patients to more complex HF units. This study will be the first to examine the clinical-epidemiological characteristics of AHF in Peru with a follow-up of 1 year after the event and will provide relevant information on these observable characteristics for the management of high-complexity patients.

## Introduction

Heart failure (HF) is a nosological entity of slow, progressive and sometimes unpredictable course, characterized by clinical, structural and functional deterioration of the heart, resulting from increased diastolic filling pressures and inadequate cardiac output unable to meet the metabolic and energetic demands of the organism (1). Globally, HF is a public health problem (2) with more than 60 million people suffering from this condition (29% have mild, 19% moderate and 51% have severe HF) in 2017. It is more frequent in females (34.8 million cases) than in males (29.5 million cases) and in high-income countries (871.1 cases per 100,000 population) (3), in which it is estimated that 1–2% of people live with HF (4). In addition, HF is estimated to have a high burden of disease, high rates of mortality and re-hospitalizations, impaired quality of life, and a significant economic burden, particularly due to frequent readmissions with a median hospital stay of 8.5 days [interquartile range (IQR: 7.38–11 days)] exceeding costs of $39 billion annually for health care systems (5–7).

In recent decades, the prognosis of patients with HF has considerably improved mainly due to significant innovations in medical therapy with neuromodulatory drugs and the development of device engineering (8). The prevalence of HF is increasing but with the development of effective treatment the survival is longer (9). Nonetheless, despite optimal medical therapy, 5% of patients with HF will inevitably progress to more advanced stages of the disease (10), being predominantly refractory with more severe signs and symptoms, more frequent decompensations and re-hospitalizations, poor quality of life and survival of <25% at 1 year and <8% at 2 years after diagnosis (11). From a health economics perspective, although advanced HF (AHF) affects only 1% of the population with HF, its high frequency of re-hospitalizations generates a significant consumption of resources that account for 60% of care costs, especially when surgical interventions such as heart transplantation or the implantation of a mechanical ventricular assist device are indicated (12). Therefore, the Interagency Registry for Assisted Mechanical Circulation (INTERMACS) classification system is a useful strategy to meet the needs of patients who are not eligible for advanced therapies, optimizing and implementing management strategies that reduce the high symptomatic burden of AHF and improving the quality of life of these end-stage patients. This system allows for better patient selection, better prognosis and determines the urgency of intervention (13).

The incidence of AHF is 0.2% of the world population (14). In the U.S., it is estimated that 250,000–500,000 patients are refractory to maximized medical therapy (7). In low- and middle-income countries, the severity of HF varies between countries and regions, with a prevalence of AHF ranging from 30.1 to 56.4% at diagnosis (15). Africa and Asia are the regions with the most severe HF phenotypes and have the lowest rate of health insurance, medication and educational level (16). In Latin America and the Caribbean, the prevalence of AHF varies between countries; between 4 and 6% of patients in Colombia have AHF with an annual mortality rate of almost 75% (17), about 2% of HF cases in Chile are refractory (18), and in Peru, 61 patients with AHF were transplanted between March 2010 and February 2018, and 26.3% of these patients required some type of mechanical circulatory assistance for bridging to cardiac transplantation (19).

In recent years, there have been reports of an increase in cardiovascular risk factors and in the survival of patients with HF in Latin American and Caribbean countries (20, 21). However, there is still little epidemiological evidence on AHF in the Peruvian population, which is corroborated by a bibliometric study on cardiovascular health in Peru, in which the main publications are related to HF in clinical trials (22). This increases the need to know the clinical and epidemiological profile and the therapeutic management of AHF in Peru in order to provide scientific evidence that is useful in clinical practice. Information on AHF in the Peruvian population is necessary and will serve as scientific evidence for the evaluation and development of strategies to reduce mortality and morbidity rates in patients with this medical condition.

## Materials and Methods

### Aim, Design and Setting of the Study

The general objective will be to determine the epidemiological, clinical, therapeutic, and annual survival characteristics of patients diagnosed with AHF treated in hospitals with HF units in the city of Lima, Peru. The specific objectives of the study will be: to determine the annual mortality of patients with AHF, calculate the rehospitalization rate of patients with AHF, determine the factors associated with a higher annual mortality in patients with AHF, correlate the INTERMACS profile with the outcomes of death, cardiac transplantation or mechanical assistance at 1 year in patients with AHF, determine the prevalence of patients with AHF in palliative therapy and with intermittent inotropic infusion, and finally, to build a prognostic score with the study variables and correlate with known scores such as the Seattle Heart Failure model.

This will be an observational, prospective, multicenter study with evaluation at baseline and follow-up at 1, 3, 6, and 12 months after study entry.

It will be performed in the cardiology services of the referral health facilities at the national level of social security, which are located in Metropolitan Lima: Instituto Nacional Cardiovascular-INCOR, Hospital Nacional Guillermo Almenara Irigoyen, Hospital Nacional Edgardo Rebagliati Martins and Hospital Nacional Alberto Sabogal Sologuren of Essalud.

### Participants and Selection Criteria

The inclusion and exclusion criteria are specified in Table 1.

**Table 1:** Selection criteria for participants to be included in the study.

Inclusion criteria	Patients over 18 years of age
	AHF defined as: (a) persistent symptoms of NYHA III - IV heart failure despite maximal optimal medical therapy (may include ICD, or CRT-D > 3 months) or the presence of medication intolerance; and (b) at least 1 of the following criteria will be included: (1) LVEF ≤ 30%, (2) isolated right HF, (3) severe inoperable heart valve disease, (4) severe inoperable congenital heart disease, (5) persistently high pro-BNP or severe left ventricular diastolic dysfunction; (c) history of more than one hospitalization (>24 h) or unplanned visit to emergency room for decompensated HF requiring high-dose intravenous diuretics (or combination) or inotropics/pressors or malignant arrhythmias in the past year; and (d) severe exercise compromise or 6MWT distance <300 meters, or peak oxygen consumption <12 ml/k/min (or <50% of predicted) of cardiac origin.
Exclusion criteria	Comorbidities with life expectancy <1-year
	Pericardial disease
	Active myocarditis
	Pregnant women
	Cardiac conditions amenable to surgical or percutaneous treatment (myocardial revascularization surgery, valve surgery or TAVI)
	Severe pulmonary disease, non-cardiac cirrhosis and end stage kidney disease (not related to HF).

*HF, heart failure; AHF, advanced heart failure; ICD, implantable cardioverter defibrillators; CRT-D, cardiac resynchronization therapy–device; LEVF, left ventricular ejection fraction; pro-BNP, pro-B-type natriuretic peptide; 6MWT, 6 minute walk test; TAVI, percutaneous aortic valve implantation*.

### Informed Consent

Patients must provide consent by means of a written, signed and dated form. This document was approved on November 17, 2021, by the Ethics Committee of the National Cardiovascular Institute INCOR with resolution N°. 46/2021-CEI.

### Study Procedures

At each institution included in the study, a cardiologist will be responsible for collecting the data during outpatient consultation or hospitalization and sending them to the registry coordinating center (Instituto Nacional Cardiovascular-INCOR) after obtaining informed consent. The data collection process will be performed on the data collection sheet designed for the study. The final database will only be accessible with an electronic user ID and password and will be stored in a secure electronic database.

For each patient included we will record the general characteristics (age, sex, care center), epidemiological characteristics (history of cardiovascular risk factors), etiology of HF, medication used (doses of the main HF medicines achieved), tests performed (echocardiography, 6-minute walk test, cardiopulmonary test, etc.), inpatient and/or outpatient treatment (intermittent ambulatory inotropic therapy, iron deficit treatment, etc.), number of hospitalizations due to decompensation or malignant arrhythmias, treatment during hospitalizations, and outcomes (death, transplantation and/or placement of mechanical assistance devices).

Follow-up data at 1, 3, 6, and 12 months after study entry will be collected by telephone interview (for remote data collection in a timely manner and taking pandemic conditions) and by reviewing patients' electronic medical records. Detailed information on the variables to be collected can be found in the Supplementary Table S1.

### Sample Size and Power

All patients attended in the centers participating in the study over the course of 1 year who meet the inclusion criteria will be included. It is known from studies in other countries that AHF is present in up to 5% of patients with HF (23). Thus, for an unknown population of patients with HF, a minimum sample of 81 patients with AHF is estimated, based on an alpha value of 0.05, a 95% confidence interval (95% CI), and an expected 10% loss to follow-up.

### Outcome Measures

Cardiovascular mortality (up to the first year after study entry). Cardiac transplantation. Time to first hospitalization for HF decompensation (requiring hospitalization with use of intravenous diuretics or inotropics).

### Statistical and Analytical Plans

Stata version 14 will be used to conduct the analyses. Clinical and demographic characteristics of the patients will be reported according to the type of variable included. Numerical variables will be reported as means and standard deviation or medians and IQR, while categorical variables will be reported by absolute frequencies and proportions.

Comparisons of predictor variables for the outcomes of interest will be performed using the chi-square test for categorical variables. For numerical variables, the Student's *t*-test or one-way analysis of variance or non-parametric test will be used depending on fulfillment of the normal distribution assumption. The risk ratio (RR) and 95% CI will be estimated using generalized linear models with binomial family and log-link function. The variables age, sex, Seattle HF model score, HF etiology, and hospitalizations will be considered as variable predictors.

In addition, the cumulative in-hospital mortality during follow-up will be estimated with the Kaplan-Meier method, and the log-rank test will be used to evaluate the differences between survival curves. Finally, Cox regression models will calculate Hazard ratios (HRs) and 95% CI.

*P*-values <0.05 will be deemed statistically significant.

### Data Storage and Management

The principal investigator (PI) (cardiologist) at the participating tertiary health centers will enter the data of the participants included in the study. A physical registration system will be used for this process. Each registered patient will be assigned a unique identification code to preserve anonymity. The physical forms will be entered into an electronic database that will be preserved with a password with which only the PI of the study will have access to the final data set of the study.

### Ethics and Dissemination

The study was approved by the Ethics Committee of the “Instituto Nacional Cardiovascular Carlos Alberto Peschiera Carrillo-INCOR”, an institutional review committee recognized by the National Health Institute of Peru (https://www.ins.gob.pe/registroEC/listaregistroCIEI.asp), with Approval Report N° 46/2021-CEI. The study will be conducted in accordance with the Declaration of Helsinki.

At the end of the study, the results will be published in articles in peer-reviewed scientific journals and at scientific congresses. To improve the quality of the reporting of the results, this study will follow the guidelines of the STROBE statement for the writing of articles generated from the study (24).

### Status and Timeline of the Study

**Figure 1:**
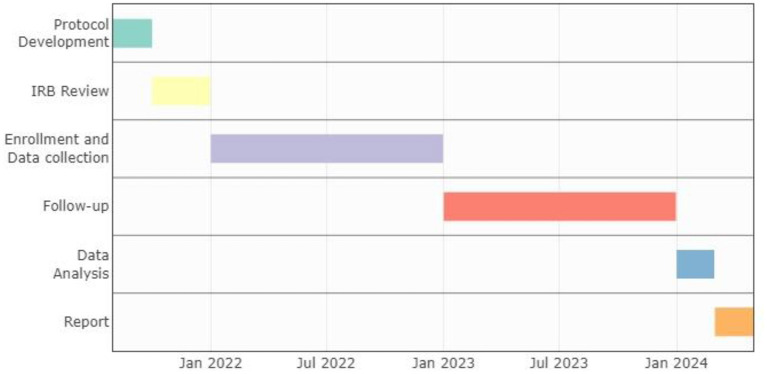
Study timeline and milestones. We use package *vistime* in RStudio for visualization.

Patient enrollment will be from January 15, 2022 to December 31, 2022 in EsSalud hospitals with trained cardiologist medical staff who provide care to patients with AHF. Once the patient is admitted to the study, telephone and/or face-to-face follow-up will be performed at 1, 3, 6, and 12 months after study entry. Thus, follow-up is expected to end in December 2023 and presentation of the final results will be in April 2024. The timeline of the study is specified in Figure 1.

## Discussion

The present study will provide information on the epidemiological, clinical, therapeutic, and annual survival characteristics of patients diagnosed with AHF treated in hospitals with HF units in the city of Lima, Peru. Therefore, we seek to increase knowledge about the epidemiology and appropriate management of AHF in high resolution hospitals in Peru, which have a high demand for medical care and where patients do not have a palliative care program when they do not have therapeutic options.

However, patients with AHF should be properly defined and identified to facilitate the application of treatments such as cardiac transplantation or long-term mechanical circulatory support devices. In Peru, there is no scientific evidence describing the current panorama of AHF in the population. This means that physicians are not properly trained with the characteristics of the Peruvian population to identify patients who could be candidates for such therapies and to recognize the optimal time to refer them to more complex HF units. Therefore, a hospital registry of AHF in the network of hospitals with HF units in the country is necessary to know the incidence, prevalence, and prognostic implications of this pathology in the Peruvian population.

Among the strengths of the study is its multicenter character, which will help to obtain a representative sample of the population with the condition studied, increasing the external validity of the results. In addition, it will be the first study to examine the epidemiological characteristics of AHF in public hospitals in Peru, providing relevant information on these observable characteristics in the management of high complexity patients. On the other hand, among the limitations of the study, the study can only describe associations and not causality due to its observational design. AHF is an infrequent pathology that will generate a low number of patients to be included in the study and there could be errors in data collection when using medical records.

Currently, the coronavirus disease pandemic (COVID-19) has led to changes in the epidemiology and management of HF worldwide, with decreased hospital admissions, delayed emergency care, and increased risk of complications (25, 26). In addition, these changes extrapolate into barriers to research, especially in the conduct of studies that require extensive physical examination, primary data collection, and face-to-face follow-up of participants (27, 28). Despite the fact that the study will be conducted in a pandemic context and with limited access to hospital centers, it is a priority to have clinical-epidemiological information on AHF in high resolution hospital centers in Peru to help Peruvian cardiologists in the search for the optimal management of these patients.

## Author Contributions

MC-D: conceptualization, project administration, resources, investigation, supervision, methodology, writing—original draft, and writing—review and editing. RL, AH-V, and RV-F: investigation, methodology, writing—original draft, and writing—review and editing. All authors contributed to the article and approved the submitted version.

## Funding

This protocol was self-funded by the authors.

## Conflict of Interest

The authors declare that the research was conducted in the absence of any commercial or financial relationships that could be construed as a potential conflict of interest.

## Publisher's Note

All claims expressed in this article are solely those of the authors and do not necessarily represent those of their affiliated organizations, or those of the publisher, the editors and the reviewers. Any product that may be evaluated in this article, or claim that may be made by its manufacturer, is not guaranteed or endorsed by the publisher.
