# Supplementary material for: Study Protocol for the Peruvian Registry of Advanced Heart Failure (REPICAV)
Source: Front Cardiovasc Med. 2022 May 31;9:896821. doi: 10.3389/fcvm.2022.896821 (PMC9194087; doi:10.3389/fcvm.2022.896821)
Supplement: Supplementary file 1 [file Data_Sheet_1.docx]

Supplementary Material

**Supplementary data.** Data collection sheet.

**HOSPITAL RECORD OF ADVANCED HEART FAILURE**

**Data collection sheet**

Case Nº:………………….

Hospital:_____________________________________

Date of entry to the study:________________

Age:______ Gender:______ Origin:_______________

**Etiology of HF:**

Ischemic ( ) Non-ischemic ( ): Valvular ( ): Aortic ( ) Mitral( ) Tricuspid ( )

Congenital ( )

Cardiomyopathy ( )

Family ( )

Chemotherapy ( )

Infectious ( )

Unknown ( )

**History:**

DM2 ( ) AHT ( ) Atrial fibrillation ( ) COPD ( ) CKD ( )

Time of diagnosis of heart failure: ……………….. (years)

**Medication on admission. Devices upon admission.**

ACE inhibitors/ARA ( ) Dose:……………….. ICDs ( )

Beta-blockers ( ) Dose:………………. ICDs + CRT ( )

Aldosterone antagonist ( ) Dose:………. Dual-chamber pacemaker ( )

ARNI ( ) Dose: ……………… Single-chamber pacemaker ( )

iSGLT2 ( ) Dose: ……………….

Furosemide ( ) Dose:………………

Another diuretic ( ) type:__________________ dose:………………

Ivabradine ( ) Dose:………………

Anticoagulation ( )

Inotropics ( )

**Current illness:**

Situation at admission: Ambulatory ( ) Hospitalized ( )

NYHA: III ( ) III b ( ) IV ( )

No. hospitalizations due to failure in the last year: __________

BMI: ______ BP:________ CR:_______

INTERMACs at income: ________

**Laboratory:**

Sodium:_______ mmol/L Potassium:_______ mmol/L Creatinine:_______

Urea:_______ GFR: __________ml/min Uric acid:________

Cholesterol:_________ pro-BNP:____________ Ca-125:________

Hb:_________ Lymphocytes:_________ GOT:_________

GPT:_________ Bilirubin T:_________ Lactate:_________

**Echocardiography.**

LVEF:__________ FS-RV:_____________ TAPSE:________

LVEDD:_________ LVESD:__________ e/e`:___________

**Quality of life:**

6MWT ( ) : distance reached: _____ m.

Grip force ( ): _____ Kg.

MLHFQ score: __________

**Initial stratification**.

Survival 1 year SHFM:________ Survival 5 year SHFM: __________

Seattle Proportional Risk Model: _________death from pump failure

**Hemodynamics. Yes ( ) No ( )**

RAP:______ mPAP:_______ PWCP:_______CO:______ CI:________

PVR:______ SVR:______ TPG:_______ DPG:_____ PAPi:______

**Cardiopulmonary test. Yes ( ) No ( )**

VO2 max :________ VCO2:___________

**FOLLOW-UP**

**Clinical and medication monitoring (dose) and devices:**

|  | **1 month** | **3 months** | **6 months** | **12 months** |
| --- | --- | --- | --- | --- |
| NYHA |  |  |  |  |
| INTERMACs |  |  |  |  |
| ACE inhibitors/ARNI |  |  |  |  |
| Beta-blockers |  |  |  |  |
| Aldosterone antagonist |  |  |  |  |
| ARNI |  |  |  |  |
| ISGLT2 |  |  |  |  |
| Ivabradine |  |  |  |  |
| Furosemide |  |  |  |  |
| Another diuretic |  |  |  |  |
| Anticoagulation |  |  |  |  |
| Inotropics |  |  |  |  |
| ICDs |  |  |  |  |
| ICDs + CRT |  |  |  |  |

**Intermittent inotropics: Yes ( ) No ( )**

Moment of entry: Hospitalized ( ) Ambulatory ( )

Maximum number of pulses:__________

Intermittent inotropic time:_________

**Iron therapy: Yes ( ) No ( )**

Basal ferritin:__________ Basal transferrin sat:__________

Ferritin 3M:____________ Transferrin sat 3M:____________

Used drug: Iron saccharate ( ) Carboxymaltose ( ) Others ( )

6MWT prior to therapy:___________

6MWT 3 months post therapy:____________

**UNLINKS**

Death: Yes ( ) No ( ) Reason: Arrhythmic ( ) Pump failure( )

Death date:______________

Transplant: Yes ( ) No ( ) Date of HT:_____________

INTERMACs to HT:________

Hospitalización Yes ( ) No ( ) Hospitalization date:___________ by decompensation Hospital stay:________days

Number of hospitalizations during follow-up ( )

Short-term LVAD ( )

Long-lasting LVAD ( )

ECMO ( )

**Supplementary Table 1. Study variables.**

|  | **VARIABLE** | **TYPE** | **DEFINITION** | **FINAL EXPRESSION** |
| --- | --- | --- | --- | --- |
| General | Age | Numeric | Age in years at the time of registration | X years |
|  | Sex | Categorical | Sex of study participant | 1= male, 2= female |
|  | Source | Categorical | Department of Peru from which it originates | 1= Lima, 2=rest of the country |
|  | Date of entry | Numeric | Date of entry into the study | Day/month/year |
| Background | Ischemic etiology | Categorical | Ischemic cause of heart failure (e.g., previous myocardial infarction) | 0= no, 1 = yes |
|  | Non-ischemic etiology | Categorical | Non-ischemia-related etiologies as a cause of heart failure | 1= Valvular, 2=Congenital, 3=Myocardiopathy, 4=Familial, 5= Chemotherapy, 5= Infectious, 6= Unknown |
|  | Type of valve disease | Categorical | Primary valvulopathy responsible for the origin of the HF (exclude secondary insufficiencies) | 1=Aortic regurgitation. 2=Aortic stenosis, 3=Primary mitral regurgitation, 4=Mitral stenosis, 5=Primary tricuspid regurgitation, 5=Tricuspid regurgitation. |
|  | Diabetes mellitus | Categorical | History of diabetes mellitus type I or II diagnosis | 0= no, 1 = yes |
|  | Atrial fibrillation | Categorical | History of a diagnosis of atrial fibrillation, regardless of the rhythm at the time of recording | 0= no, 1 = yes |
|  | Time to Diagnosis of Heart Failure | Numerical, continuous | Years elapsed from diagnosis of heart failure until study entry | In years completed |
|  | ICDs alone | Categorical | Patient with implantable defibrillator | 0=no, 1= yes |
|  | ICDs + Resynchronizer | Categorical | Patient with implantable defibrillator with resynchronization device | 0=no, 1= yes |
|  | Situation at admission | Categorical | Patient's condition at study entry | 0= Hospitalize, 1= outpatient |
|  | NYHA functional class | Categorical, ordinal | Functional status of dyspnea, fatigue according to NYHA classification. (1) | 1= NYHA III, 2=NYHA IIIb, 3=NYHA IV |
|  | Hospitalizations in the last year | Numeric | Number of hospitalizations for heart failure during the last year prior to the start of the study (if hospitalized, include the current one). | In whole numbers |
| Current | 6-minute walk test | Continuous numerical | Distance traveled in the standardized 6-minute walk test | In meters |
|  | Hand grip strength | Continuous numerical | Value in kilograms obtained by measuring the hand grip force with a dynamometer. | In kilograms |
|  | Body mass index | Continuous numerical | Value resulting from dividing the weight by the square of the patient's height. | In Kg/m^2^ |
|  | Systolic blood pressure | Continuous numerical | Systolic blood pressure value at study entry | In mmHg |
|  | Cardiac frequency | Continuous numerical | Cardiac frequency value at study entry | In beats per minute |
|  | Serum sodium | Continuous numerical | Blood sodium value at study entry | In mmol/L |
|  | Serum potassium | Continuous numerical | Blood potassium value at study entry | In mmol/L |
|  | Serum creatinine |  |  |  |
|  | Estimated glomerular filtration rate | Continuous numerical | Value found using Cockroft-Gault formula | In ml/min |
|  | Uric acid | Continuous numerical | Blood uric acid value at study entry | In mg/dl |
|  | Cholesterol | Continuous numerical | Total cholesterol blood value at study entry | In mg/dl |
|  | NT-PROBNP | Continuous numerical | Blood value of total NT-PROBNP at study entry | In pg/ml |
|  | Ca 125 | Continuous numerical | Total Ca 125 blood value at study entry | U/ml |
|  | Hemoglobin | Continuous numerical | Hemoglobin value at study entry | In mg/dl |
|  | GOT | Continuous numerical | GOT value at entry | In mg/dl |
|  | GPT | Continuous numerical | GPT value at entry | In mg/dl |
|  | Lymphocytes | Continuous numerical | Percentage value of lymphocytes in the leukogram at study entry | In percentage |
| Echocardiogram | LV ejection fraction | Numerical, continuous | Percentage value of left ventricular ejection fraction measured by echocardiography closest to the time of study entry. | In percentage |
|  | RV shortening fraction | Numerical, continuous | Percentage value of the right ventricular shortening fraction measured by echocardiography closest to the time of study entry. | In percentage |
|  | LVEDD | Continuous numerical | Value in millimeters of the diameter in diastole of the left ventricle taken in the parasternal long axis. | In millimeters |
|  | LVESD | Continuous numerical | Value in millimeters of left ventricular systolic diameter taken in long parasternal axis. | In millimeters |
|  | Relation e/e´ | Continuous numerical | Ratio of mitral flow E-wave velocity to tissue Doppler E' wave over the mitral annulus | In numerical value |
| Stadium at the entrance | INTERMACS at time of entry | Categorical | Patient classification according to INTERMACS profile | 1= INTERMACS 1: Cardiogenic shock  2=INTERMACS 2: progressive deterioration  3=INTERMACS 3: Inotropic dependency  4=INTERMACS 4: symptoms at rest, oral therapy at home  5=INTERMACS 5: Exercise Intolerance  6=INTERMACS 6: Exercise limitation  7=INTERMACS 7: advanced NYHA III |
| Maximum stage | INTERMACS maximum achieved | Categorical | Patient classification according to the maximum INTERMACS profile reached during the study. | 1=INTERMACS 1: Cardiogenic shock  2=INTERMACS 2: progressive deterioration  3=INTERMACS 3: Inotropic dependency  4=INTERMACS 4: symptoms at rest, oral therapy at home  5=INTERMACS 5: Exercise Intolerance  6=INTERMACS 6: Exercise limitation  7=INTERMACS 7: advanced NYHA III |
| Quality of life | MLHFQ | Numerical, continuous | Value obtained by the patient in the Minnesota questionnaire at the time of study entry. | In decimal numbers |
| Risk models | Survival 1 SHFM | Numeric | Percentage survival at one year according to the Seattle score at study entry. | In percentage |
|  | Survival 5 SHFM | Numeric | Percentage of 5-year survival according to the Seattle score at study entry. | In percentage |
|  | Seattle proportional risk model | Numeric | Percentage of annual mortality due to arrhythmic causes, calculated at study entry. | In percentage |
| Hemodynamics | RAP | Numeric | Mean right atrial pressure of the right atrium from the closest catheterization | In mmHg |
|  | mPAP | Numeric | Mean pulmonary artery pressure of the nearest catheterization site | In mmHg |
|  | PWCP | Numeric | Pulmonary artery occlusion pressure or capillary pressure. | In mmHg |
|  | CO | Numeric | Cardiac output obtained by thermodilution | In L/min |
|  | CI | Numeric | Cardiac output indexed to body surface area | In L/min/m^2^ |
|  | PVR | Numeric | Pulmonary vascular resistance | In Wood units |
|  | SVR | Numeric | Systemic vascular resistance | In dynas/s/cm^2^ |
|  | Transpulmonary gradient | Numeric | Difference between mean pulmonary BP and capillary pressure. | In mmHg |
|  | PDG | Numeric | Difference between pulmonary diastolic BP and capillary pressure. | In mmHg |
|  | PAPi | Numeric | Pulmonary artery pulsatility index (SPAP-DPAP)/RAP |  |
| Cardio Pulmonary Test | VO_2_ max | Numeric | Maximal oxygen consumption obtained during the cardiopulmonary test | In ml/kg/min |
|  | VCO_2_ | Numeric | Amount of carbon dioxide exhaled per time in cardiopulmonary test | In ml/min |
| Medication | Beta-blockers | Categorical | Beta-blocker user patient | 0=no, 1=yes |
|  | ACE inhibitors | Categorical | Patient receiving angiotensin converting enzyme inhibitors. | 0=no, 1= yes |
|  | ARA2 | Categorical | Patient receiving angiotensin 2 receptor blockers. | 0=no, 1= yes |
|  | ARNI | Categorical | Patient user of sacubitril/valsartan | 0=no, 1= yes |
|  | Aldosterone antagonist | Categórica | Patient receiving spironolactone | 0=no, 1= yes |
|  | Diuretics | Categorical | Patient with prescription for diuretic use | 0=no, 1= yes |
|  | Furosemide dosage | Numeric | Total daily doses of furosemide or equivalent | in mg/day |
|  | Sequential lock | Categorical | Use of another diuretic in addition to LD and aldosterone antagonist | 1=yes, 2= no |
|  | 3rd diuretic | Categorical | Type of diuretic in case of sequential blockade | 1= Thiazidic, 2=acetazolamide |
|  | ISGLT2 | Categorical | SGLT2 inhibitor user patient | 0=no, 1= yes |
|  | Anticoagulation | Categorical | Use of oral anticoagulants | 0=no, 1= yes |
|  | ICDs | Categorical | Patient receives an implantable defibrillator during the study | 0=no, 1= yes |
|  | ICDs + CRT | Categorical | Patient receives an implantable defibrillator with resynchronizer during the study | 0=no, 1= yes |
| Inotropic pulse therapy | Inotropic pulse | Categorical | Patient on inotropic pulse therapy (levosimendan or dobutamine) | 0=no, 1= yes |
|  | Moment of entry | Categorical |  | 0=hospitalized, 1= ambulatory |
|  |  |  |  |  |
|  | Number of sessions | Numeric |  | In number of sessions |
|  | Time in intermittent inotropia | Numeric |  | In weeks |
| IV iron | IV iron therapy | Categorical | Patient used intravenous iron therapy | 0=no, 1= yes |
|  | Drug | Categorical | Drug used for iron replacement | 0= carboxymaltose, 1=iron saccharate |
|  | 6MWT initial | Numerical, continuous | Before starting iron therapy | In meters |
|  | 6MWT final 3M | Numerical, continuous | At 3 months post iron therapy | In meters |
|  | Ferritin | Numerical, continuous | Serum ferritin value at study entry. | in mg/dl |
|  | Transferrin saturation | Numerical, continuous | Saturation value of transferrin at study entry | In percentage |
|  | Ferritin | Numerical, continuous | Serum ferritin value 3 months post therapy | In mg/dl |
|  | Transferrin saturation | Numerical, continuous | Transferrin Saturation Value 3 months post therapy | In percentage |
| Outcomes |  |  |  |  |
| Death | Death date | Numerical | Death date | Day/month/year |
|  | Death from pump failure | Categorical | Death after progressive deterioration heart failure requiring increased therapy. | 0=no, 1=yes |
|  | Arrhythmic death | Categorical | Unexpected death in a previously stable patient within 24 hours of symptom onset. | 0=no, 1=yes |
| Transplant | Heart transplant | Categorical | Patient who undergoes a heart transplant | 0=no, 1=yes |
|  | HT date | Numerical |  | Day/month/year |
| Hospitalization | Hospitalization for decompensation | Categorical | Hospitalization> = 24 h, due to decompensated HR requiring IV inotropics, high-dose IV diuretics or devices | 0=no, 1=yes |
|  | date of hospitalization | Numerical |  |  |
|  | Hospital stay | Numerical | Days of hospital stay | In days |
|  | Number of hospitalizations | Numerical | Number of hospitalizations during the study period | In integer values |
|  | Short-term LVAD | Categorical | Use of LV or RV mechanical assistance (Centrimag) | 0=no, 1=yes |
|  | Long-lasting LVAD | Categorical | Use of long-term mechanical assistance (Heart Mate III, etc.) | 0=no, 1=yes |
|  | ECMO | Categorical | Use of extracorporeal membrane oxygenation | 0=no, 1=yes |
|  | Survival | Numerical | Time between diagnosis and death | In days |

HF, heart failure; NYHA, New York Heart Association; NT-PROBNP, N-terminal (NT)-proBNP; GOT, Glutamic oxaloacetic transaminase; GPT, glutamic pyruvic transaminase; 6MWT, 6 minute walk test; LV, Left Ventricle; RV, Right Ventricle.
